# Inadequate Coping Strategies of Men who Have Committed Sexual Aggression Against Women: A Study of Their Developmental Antecedents

**DOI:** 10.1177/10790632231210534

**Published:** 2023-11-07

**Authors:** Alexandre Gauthier, Caroline Deli, Etienne Garant, Jean Proulx

**Affiliations:** 1 School of Criminology, 5622 University of Montreal, Montreal, QC, Canada; 2 Institut national de psychiatrie légale Philippe-Pinel, Montreal, QC, Canada; 3 International Centre of Comparative Criminology, 5622 University of Montreal, Montreal, QC, Canada

**Keywords:** men who have committed sexual aggression, coping strategies, sexual deviant fantasies, childhood victimizations, psychological problems, structural equation modeling

## Abstract

Several researchers have found that men who have committed sexual aggression have inadequate coping strategies (e.g., paraphilic sexual fantasies, substance abuse). However, very few researchers have empirically examined the factors potentially associated with the development of these strategies. In 2011, Maniglio hypothesized that the inadequate coping strategies of men who have committed sexual aggression are the result of childhood victimization, mediated by internalized psychological problems. The present study therefore empirically tested this hypothesis in a Canadian sample of 205 men who had committed sexual aggression against women, of whom 37 committed sexual murder. Structural equation modeling (SEM) resulted in the identification of several direct and indirect trajectories leading from childhood victimization (psychological, physical, sexual) to the development of inadequate coping strategies (paraphilic sexual fantasies, alcohol and drug use) mediated by internalized psychological problems (e.g., anxiety, depression, social isolation). The theoretical and clinical implications of these developmental trajectories are discussed.

## Introduction

Leitenberg and Henning (1995) define sexual fantasies as mental representations (e.g., thoughts, images, dialogues) that have the capacity to elicit a state of sexual excitement. When the content of these fantasies involves non-traditional sexual practices (e.g., sadomasochism) or practices that are prohibited by law (e.g., involving a non-consenting adult, or a minor), they are termed “paraphilic^1^” (Aylwin et al., 2005; Gee et al., 2004). The paraphilic sexual fantasies most frequently reported in studies of the general population involve sexual coercive acts (Arndt et al., 1985; Byers et al., 1998; Crépault & Couture, 1980; Dawson et al., 2016; Greendlinger & Byrne, 1987; Holvoet et al., 2017; Hunt, 1974; Joyal et al., 2015; Leitenberg & Henning, 1995; Miller & Simon, 1980; Person et al., 1989; Richters et al., 2003, 2008; Rye & Meaney, 2007; Sue, 1979; Williams et al., 2009) and sadistic acts (i.e., acts focused on domination, and the physical and psychological pain of partners) (Ahlers et al., 2011; Arndt et al., 1985; Crépault & Couture, 1980; Greendlinger & Byrne, 1987; Holvoet et al., 2017; Joyal et al., 2015; Person et al., 1989; Stefanska et al., 2022; Williams et al., 2009). For example, 68% of a sample of 103 academics in the United States reported having sexual coercive fantasies and 62% reported having sadistic fantasies (Williams et al., 2009). Similarly, respondents to an online survey of 1516 Canadian adults (men = 717; women = 799) reported the following sexual fantasies: domination of others (59.6%), bondage of others (48.4%), spanking or whipping of others (43.5%), abusing a drunk, sleeping, or unconscious person (22.6%), and sexual coercive acts (22%) (Joyal et al., 2015). In a recent study of 138 members of the general population of the United Kingdom (female = 83; male = 55), sexual fantasies of dominating a person were reported by 34% of participants, of spanking or whipping a person by 15.1%, and of tying a person up by 11.3% (Stefanska et al., 2022).

### Characteristics of Men Who Act Out Paraphilic Sexual Fantasies

Although high prevalence rates of coercive and sadistic sexual fantasies are found in the general population, several studies (Herbernick et al., 2017; Holvoet et al., 2017; Hunt, 1974; Janus & Janus, 1993; Joyal & Carpentier, 2017; Långström & Hanson, 2006; Långström & Seto, 2006; Oliveira Junior & Abdo, 2010; Richters et al., 2008) have reported much lower prevalence rates of fantasy fulfillment among men in the general population. For example, in Australia, Richters et al. (2008), who surveyed 16,779 adults (51.4% men) about their sexual practices, reported that only 2.2% of the men in their sample had engaged in sadomasochistic sexual practices. In Canada, similar results were reported by Joyal and Carpentier (2017), who surveyed 1040 adults (45.6% men): 6.3% of men in their sample reported engaging in behaviors of a sadistic nature at least once in their lifetime. In Belgium, Holvoet et al. (2017), who surveyed 1027 adults (44.6% male) about their sexual interests and behaviors, reported that although 26% of their sample reported an interest in sadomasochistic practices, only 7.6% had engaged in such sexual practices in their lifetime. These studies highlight that only a small percentage of men in the general population translate their sexual fantasies of a coercive or sadistic nature into actual behavior. It should be noted that the aforementioned studies, focusing on the content of sexual fantasies in the general population, did not take into account factors such as intensity, frequency, and preferences. It is conceivable that if these aspects had been considered, the percentages of sexual fantasies translating into behaviors could have been higher.

Who are the men who do act out their coercive and sadistic sexual fantasies? They can be divided into two categories based on the context in which they act out their fantasies. The first category comprises men who carry out their coercive or sadistic sexual fantasies in a consensual sexual setting, namely BDSM (bondage, discipline, domination, submission, sadomasochism), which implies that sexual pleasure is shared by both parties (the dominant and the dominated) (Pitagora, 2013). In these situations, sexual practices are performed in a well-defined sexual context, in which both parties (the dominant and the dominated) engage in role-playing dictated by a previously developed sexual script (Weinberg et al., 1984) which includes, among other things, recognition of each party’s boundaries. The second category comprises men who carry out their coercive or sadistic sexual fantasies in a non-consensual sexual context (sexual assault).

Many researchers have examined the psychosocial (e.g., demographic, psychological, psychopathological) profile of men who practice BDSM (Brown et al., 2020; Connolly, 2006; Cross & Matheson, 2006; De Neef et al., 2019; Hébert & Weaver, 2014; Higgs et al., 2021; Monteiro Pascoal et al., 2015; Paarnio et al., 2022; Richters et al., 2008; Wismeijer et al., 2013). The majority of studies have reported that men who practice BDSM have a high level of education (Connolly, 2006; Hébert & Weaver, 2014; Wismeijer et al., 2013). Wismeijer et al. (2013) reported that a college education was more common among BDSM practitioners in their sample than among the general population (70.1% vs. 34%). The psychological profile of male BDSM practitioners has been reported to be normal, if not healthier than that of the general population. For example, Hébert and Weaver (2014) reported that male BDSM practitioners fall within the normal range on the following HEXACO-60 scales (Ashton & Lee, 2009): honesty-humility; emotionality; extraversion; agreeableness; and conscientiousness. Finally, with regard to psychopathology, BDSM practitioners exhibit mental health comparable to the general population (Brown et al., 2020; Connolly, 2006; Cross & Matheson, 2006; Hébert & Weaver, 2014; Higgs et al., 2021; Monteiro Pascoal et al., 2015; Richters et al., 2008; Wismeijer et al., 2013). Richters et al. (2008) reported that psychological distress (e.g., anxiety, sadness, nervousness) was not higher in the male BDSM practitioners in their sample than in the general population. Monteiro Pascoal et al. (2015) observed significantly lower levels of distress related to sexual desire in the male BDSM practitioners in their Portuguese sample than in the general population.

**Figure 1.**
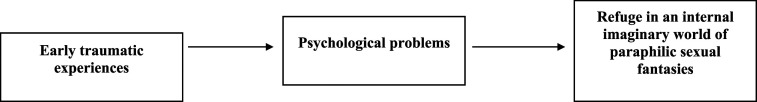
Maniglio’s (2011) model.

The results summarized above indicate that men who act out their coercive or sadistic sexual fantasies in consensual sexual settings are psychosocially normal or even healthier than the general population (e.g., high level of education, healthy personality, absence of psychopathologies). But what about men who act out these same fantasies in non-consensual sexual settings? In 2011, Maniglio advanced the following model (Figure 1) of the genesis of paraphilic sexual fantasies in men who have committed sexual aggression:

… it is possible that, in sexual offenders, early traumatic experiences, especially child sexual abuse, might result in psychological problems, in terms of either psychiatric symptoms or disorders or painful mental states; these psychological problems, in turn, might lead sexual offenders lacking effective coping strategies to take refuge in an internal, imaginary world of deviant sexual fantasy, in which they may overcome the pain, sorrow, and trouble of reality and achieve the gratification, satisfaction, and pleasure absent from reality. (p. 753)

### Negative Emotional States and Paraphilic Sexual Fantasies

Although to our knowledge, no comprehensive empirical study of Maniglio’s model (2011) has been conducted, several studies suggest an association between negative affective states and paraphilic sexual fantasies in men who have committed sexual offenses against women. For example, McKibben et al. (1994) observed that negative affective states (e.g., rejection, feelings of inadequacy, anger, humiliation, loneliness) were all positively associated with invasive paraphilic sexual fantasies. Similarly, Proulx et al. (1996) reported a positive association between certain negative affective states and the development of invasive paraphilic sexual fantasies in men who had committed sexual aggression against women or children; the most frequently reported negative affective states were anger, loneliness, and humiliation among men who had committed sexual aggression against women and who had invasive paraphilic sexual fantasies, and humiliation and loneliness among men who had committed sexual aggression against children. In 1999, Looman replicated the study of Proulx et al. (1996) and found similar results, namely that both loneliness and depression were positively associated with the development of paraphilic sexual fantasies in men who had committed sexual aggression against children. More recently, other researchers have proposed a model of fatal and non-fatal sexual aggression (Higgs et al., 2017; Higgs & Stefanska, 2018), based on data suggesting that among individuals who develop paraphilic sexual fantasies and eventually act on these fantasies in the commission of a sexual crime, paraphilic fantasies represent part of a pattern of internalised difficulties in response to adverse childhood experiences, in contrast to men who commit sexual aggression without having paraphilic sexual fantasies, for whom responses to adverse childhood experiences manifest in more externalised problems.

### Personality Traits and Negative Emotional States

While the studies described above observed a positive association between certain negative affective states and the presence of paraphilic sexual fantasies, other studies (Brittain, 1970; Curnoe & Langevin, 2002; Gauthier, 2022; Gauthier & Proulx, 2022; Lussier et al., 2001; MacCulloch et al., 1983; Proulx, 2001; Proulx & Beauregard, 2014; Proulx et al., 2007) have focused on the personality traits of men who have committed sexual aggression and who report having paraphilic sexual fantasies and negative affective states. In 1970, Brittain described the individual who commits a sadistic sexual murder as someone who tends to be socially isolated, possess low self-esteem, and feel inferior to others, and who has sadistic sexual fantasies whose function is to compensate for the lack of power in his real life. A decade later, MacCulloch et al. (1983) suggested that the sense of emptiness and inferiority mentioned by Brittain (1970) facilitates the emergence of paraphilic sexual fantasies in sadists. In their view, paraphilic sexual fantasies emerge at the beginning of adolescence and result from successive relational failures (e.g., rejection by peers and by their desired sexual object). Therefore, to fill the relational void that they experience and manage their unexpressed frustration, some adolescent victims of rejection take refuge in paraphilic sexual fantasies. These paraphilic sexual fantasies presumably allow the adolescent to reduce emotional distress (e.g., frustration, anger, anxiety) and to achieve sexual excitement and pleasure.

Several studies that have examined the psychosocial profile of men who had committed sadistic sexual aggression have reported results that partially support MacCulloch et al.’s (1983) hypothesis. For example, Proulx et al. (2007) examined the developmental, sexological, and criminological characteristics of 141 men who had committed sexual aggression against women (43 sadists and 98 nonsadists) and found that men who had committed sadistic sexual aggression not only had more paraphilic sexual fantasies than men whose sexual aggression was not sadistic, but also exhibited a high prevalence of schizoid (sadistic: 44.6%; nonsadistic:14.3%), avoidant (33.3% vs.18.6%), and dependent (46.7% vs. 38.6%) personality features, all of which are characterized by negative affective states such as humiliation, depression, and anxiety. More recently, Gauthier (2022) observed that men who had committed sadistic sexual aggression scored higher on the MCMI-I avoidant scale than men whose sexual aggression was not sadistic, were more likely to have violent paraphilic sexual fantasies (including the victim or another woman), and experienced negative affective states (e.g., anger) during the hours prior to their offense.

In parallel with these studies, which found a positive association between certain personality traits (schizoid, avoidant) and negative affective states (loneliness, anxiety, humiliation, anger) on the one hand, and the development of paraphilic sexual fantasies on the other, some researchers (e.g., Cortoni & Marshall, 2001; Feelgood et al., 2005; Looman, 1995; Marshall et al., 1999; Neidigh & Tomiko, 1991; Ward et al., 2006; Ward & Hudson, 2000) have hypothesized that sexuality (e.g., paraphilic sexual fantasies) serves to regulate negative affect in men who commit sexual aggression. This hypothesis is, for example, one of the premises of Ward et al.’s (2006) Self-Regulation Model (SRM). These authors suggest that individuals who engage in passive and active avoidance coping use paraphilic sexual fantasies to regulate their negative affective states. In addition, Marshall et al. (1999) suggested that the use of sexuality as an emotional self-regulation strategy among men who commit sexual aggression against women reflects deficient alternative coping strategies.

Sexuality (e.g., paraphilic sexual fantasies, pornography, compulsive masturbation) is not the only inappropriate coping strategy relied upon by men who have committed sexual aggression: Several models of sexual aggression (e.g., Pithers et al., 1983; Ward et al., 1998) have suggested that men who have commit sexual aggression also rely on substance use to self-regulate their negative affect. For example, Pithers et al.’s (1983) Relapse Prevention Model (RPM) suggests that men who commit sexual aggression use alcohol to cope with their negative affective states and paraphilic sexual fantasies. Ward and colleagues (Ward et al., 1998, 2004) argue that men who have committed sexual aggression following the active avoidance path of the SRM model (i.e., actively seek to avoid acting out) adopt inadequate and ineffective coping strategies, such as drinking, which disinhibit their deviant sexual desires (paraphilic sexual fantasies), which in turn increases their likelihood of acting out (sexual offending).

The objectives of this study were:

1. To empirically test Maniglio’s (2011) hypothesis that childhood sexual, psychological, and physical victimization favors the development of an anxiety-suffering personality profile in adulthood, which in turn contributes to the development of paraphilic sexual fantasies as a coping strategy in men who have committed sexual aggression against women.
2. To examine whether childhood sexual, psychological, and physical victimization also contributes—through an anxiety-suffering personality profile—to the development of other maladaptive coping strategies, such as drug and alcohol abuse, in men who commit sexual aggression against women.

## Methodology

### Sample

The participants in this study were selected from a sample consisting of 562 men who received a sentence of two years or more between the years 1995 and 2000 in Quebec for committing at least one sexual crime with physical contact. Among these 562 men, only those who committed a sexual crime with physical contact against a woman (16 years and older) (*n* = 205) were included. Thus, our sample consisted of 205 men who had committed sexual assault against a woman (37 men who had committed a sexual homicial, 168 men who had committed a non-homicidal sexual aggression). The majority of participants were French-speaking (88%), White (85.9%), single (53.9%), and unemployed (60.7%). The mean age of participants was 33.6 years (*SD* = 9.1). Regarding the criminal background of the participants, the average number of charges for sexual crimes per participant was 2.2 (*SD* = 2.08), ranging from 1 to 14 sexual crimes. Additionally, the average number of charges for violent crimes per participant was 5.1 (*SD* = 7.68), with a range of 1 to 65 violent crimes.

### Ethical Approval

The present study used the CRR database, which was established by the last author and received approval from the Ethics and Research Committee of the Correctional Service of Canada in 1994 (reference number 1435-1).

### Data Collection

The majority of the participants in this study were in the Regional Reception Centre (RRC), a federal penitentiary (Canada), at the time of their initial assessment. During their approximately 6-week stay at this institution, the participants were assessed by a multidisciplinary team consisting of psychologists, criminologists, sexologists, vocational training professionals, and correctional officers. This evaluation was conducted both for the purposes of the study and to determine the appropriate treatment and level of security for each offender prior to their transfer to another institution. Participants who had committed a sexual homicide were recruited from all Canadian penitentiaries located in Quebec. All participants (*N* = 205) signed a consent form that stipulated that all information collected would be used strictly for research purposes. Each participant completed a series of psychometric tests and participated in several semi-structured interviews based on the Computerized Sex Offender Questionnaire (CSOQ; St-Yves et al., 1994). The CSOQ collects information on several facets of the lives of men who have committed acts of sexual aggression (e.g., work, family, school, criminal and correctional files). Information gathered in the interviews was supplemented with official information (e.g., police reports, victim-impact statements). When official information did not match the information provided by the participant, the official information took precedence. Finally, regarding the CRR database, inter-rater reliability tests were conducted during its creation in 2000, by the team responsible for collecting data, on 92 dichotomous variables out of the 2500 variables it contains. The mean Cohen’s kappa was .86 (*SD* = .18), indicating almost perfect agreement (Landis & Koch, 1977).

### Measures

#### Deviant Sexual Fantasies

The presence of paraphilic sexual fantasies was coded dichotomously (0 = absent; 1 = present) on the basis of the following question: Do you have violent paraphilic sexual fantasies toward women? The majority of participants in the current study underwent a phallometric assessment during their initial evaluation, and the results of this assessment were discussed with a forensic psychologist. This enhanced the reliability of the information provided by the participants.

#### Childhood Victimization

The presence of sexual, physical, and psychological (emotional) victimization experienced as a minor (before age 18) was coded dichotomously (0 = absent; 1 = present).

#### Alcohol use Problems

Variables related to problem drinking were coded dichotomously (0 = absent; 1 = present) on the basis of DSM-IV criteria. The variables that were examined were: regular alcohol use, work-related problems (problems at work caused by alcohol consumption), and alcohol dependence.

#### Drug-use problems

Variables related to drug-use problems were coded dichotomously (0 = absent; 1 = present) on the basis of DSM-IV criteria. The variables that were examined were: drug treatment, drug dependence, and social problems due to drug use.

#### Personality Profile

Each participant’s personality profile was assessed using the French version of the Millon Clinical Multiaxial Inventory (MCMI-I; Millon, 1983), which has been validated in a French-speaking Quebec sample (Landry et al., 1996). The MCMI-I is a questionnaire composed of 175 true-or-false questions. The answers obtained are compiled to obtain a raw score for each of the following 11 personality disorders: schizoid, avoidant, dependent, histrionic, narcissistic, antisocial, obsessive-compulsive, passive-aggressive, schizotypal, borderline and paranoid. These raw scores are then transformed into base rates, which are interpreted as follows: A base rate greater than 74 and less than 85 indicates the presence of characteristics specific to a disorder (trait), while a base rate of at least 85 indicates the marked presence of these characteristics (disorder).

Factor analyses of the MCMI-I scales associated with anxiety-suffering personality profiles (e.g., avoidant, dependent, borderline, passive-aggressive) revealed that only the factor composed of the avoidant, passive-aggressive, and borderline scales had an adequate Kaiser-Meyer-Olkin test value (KMO = .83; Dodge, 2008). Therefore, in this study, only the avoidant, passive-aggressive, and borderline scales of the MCMI-I were further analysed.

### Analytical Strategy

First, descriptive analyses of demographic variables were performed, using SPSS version 27 (Table 1). In a second step, data from all participants (*N* = 205) regarding childhood victimization (3 variables), problematic alcohol use (3 variables), problematic drug use (3 variables), personality profile (3 variables), and paraphilic sexual fantasies (1 variable) were exported from SPSS to Mplus V8 software 4 (Muthén & Muthén, 2017) in order to perform multiple imputation and structural equation modeling analyses.

**Table 1.** Descriptive Statistics: Childhood Victimization, Anxious-suffering Personality, Drug problems, Alcohol problems, and Paraphilic sexual fantasies.

Variables	Total sample (*N* = 205)
Childhood victimization (before age 18)	
Sexual victimization	35%
Physical victimization	51%
Psychological victimization	54.4%
Anxious-suffering personality (MCMI-1)	
Avoidant personality scale	*M* (62.81) *SD* (27.16)
Passive-aggressive personality scale	*M* (52.59) *SD* (29.03)
Borderline personality scale	*M* (58.36) *SD* (15.38)
Avoidant personality scale ≥ 75	34.7%
Passive-aggressive personality scale ≥ 75	27.7%
Borderline personality scale ≥ 75	13.5%
Drug problems (DSM-IV)	
Drug treatment	20.9%
Drug dependence	40.8%
Social problems	38.3%
Alcohol problems (DSM-IV)	
Regular consumption	71.4%
Work-related problems	23.8%
Alcohol dependence	42.2%
Paraphilic sexual fantasies	27.2%

Multiple imputation is a statistical technique used to compensate for missing data (Cox et al., 2014; Enders & Mansolf, 2018; van Ginkel et al., 2020). Enders and Mansolf (2018) report that it is the best technique for managing missing data without affecting the quality of analyses. According to those authors, this technique allows analyses of acceptable quality to be conducted in a sample of 200 participants with a missing-data rate of 40%. In this study, the missing-data rate for the MCMI-I data was 27%.

Structural equation modeling is a statistical technique “used to represent and test series of relationships between observed and latent variables” (Longpré et al., 2018, p. 289). Model selection is based on the following fit indices (McDonald & Ho, 2002): the root-mean-square error of approximation (RMSEA); the comparative fit index (CFI); and the Tucker-Lewis index (TLI). Xia and Yang (2019) describe the RMSEA as an absolute fit index that assesses the difference between the fit of the model in question and that of a perfect model. Both CFI and TLI have been described as incremental indices that compare the fit of the model in question to the model with the worst fit. RMSEA values below .05 are considered “excellent”, and values between .05 and .08 are considered “acceptable” (Wang & Wang, 2019, p. 21). For CFI and TLI, values exceeding .95 are considered “excellent” and values exceeding .90 are considered “acceptable” (Schumacker & Lomax, 2016). In addition, we used the weighted least-squares estimator of the adjusted mean and variance (WLSMV), which provides the best option for modeling continuous and categorical data (Kline, 2016). Finally, it is important to emphasize that structural equation modeling (SEM) does not prove the existence of a causal relationship, but only supports the idea that such a relationship exists (Bollen & Pearl, 2013).

## Results

### Developmental Model of Deviant Sexual Fantasies

The first developmental model that we identified focuses exclusively on the development of paraphilic sexual fantasies in men who commit sexual assault against a woman. This model is a replication of the conceptual model proposed by Maniglio (2011). It consists of three independent manifest variables, a dependent manifest variable, and a latent factor. The independent manifest variables were physical, psychological, and sexual victimization, and the dependent manifest variable was paraphilic sexual fantasies. The latent factor, namely *anxious-suffering personality* was composed of three continuous manifest variables (derived from MCMI-I avoidant, passive-aggressive, and borderline personality scores).

**Figure 2.**
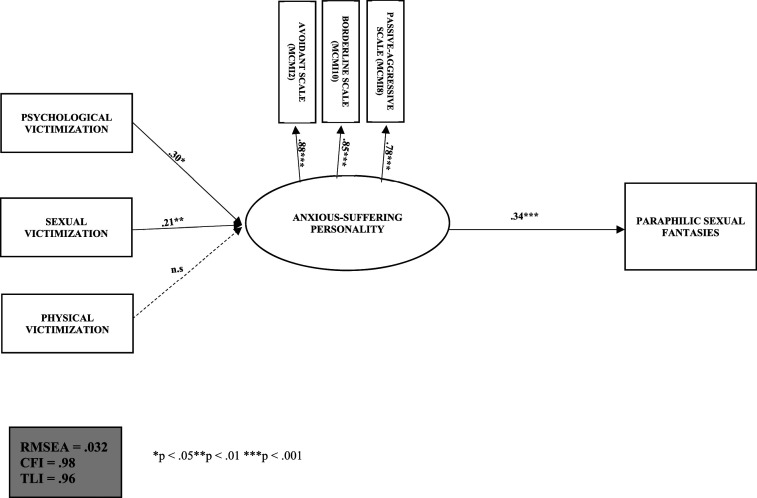
Anxiety-suffering personality profile as a mediator of childhood victimization and the development of paraphilic sexual fantasies among men who sexually assaulted women (*N* = 205).

The fit indices of the model (Figure 2) were excellent: RMSEA = .032, CFI = .98 and TLI = .96. Two indirect trajectories were identified: 1) childhood psychological victimization contributes (β = .30, *p* < .05) to the development of an anxious-suffering personality profile in adulthood, which, in turn, contributes (β = .34, *p* < .001) to the development of paraphilic sexual fantasies; 2) childhood sexual victimization contributes (β = .21, *p* < .01) to the development of an anxiety-suffering personality profile in adulthood, which, in turn, contributes (β = .34, *p* < .001) to the development of paraphilic sexual fantasies. Physical victimization experienced during childhood did not significantly contribute to the development of an anxiety-suffering personality profile in adulthood.

### Developmental Model of Inadequate Coping Strategies

Our developmental model of inadequate coping strategies in men who commit sexual aggression consists of three independent and one dependent manifest variable, and three latent factors. The independent manifest variables were physical, psychological, and sexual victimization, and the dependent manifest variable was paraphilic sexual fantasies. The three latent factors were: 1) anxious-suffering personality, which was composed of three continuous manifest variables (derived from MCMI-I avoidant, passive-aggressive, and borderline personality scores); 2) alcohol, which was composed of three dichotomous manifest variables (regular alcohol use, work-related problems, and alcohol dependence); and 3) drugs, which was composed of three dichotomous manifest variables (drug treatment, drug dependence, and social problems due to drug use).

**Figure 3.**
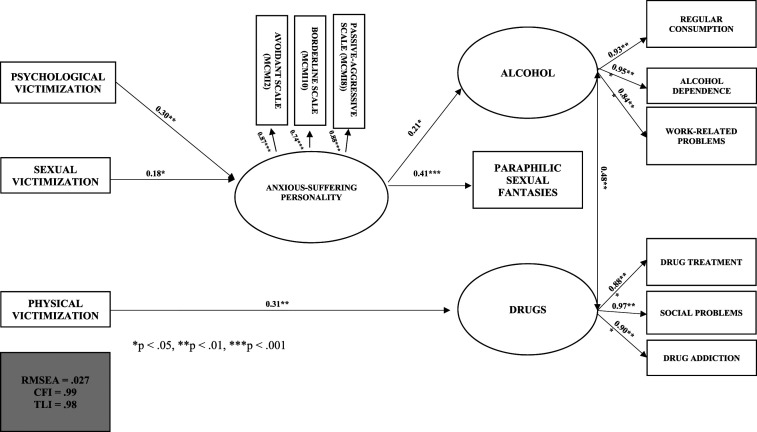
Anxiety-suffering personality profile as a mediator of childhood victimization and the development of inadequate coping strategies among men who sexually assaulted women (*N* = 205).

The fit indices of the model (Figure 3) were excellent: RMSEA = .027, CFI = .99 and TLI = .98. Four indirect trajectories and one direct trajectory were identified: 1) childhood psychological victimization contributes (β = .30, *p* < .01) to the development of an anxious-suffering personality profile in adulthood, which, in turn, contributes (β = .41, *p* < .001) to the development of paraphilic sexual fantasies; 2) childhood sexual victimization contributes (β = .18, *p* < .05) to the development of an profile in adulthood, which, in turn, contributes (β = .41, *p* < .001) to the development of paraphilic sexual fantasies; 3) childhood psychological victimization contributes (β = .30, *p* < .01) to the development of an anxiety-suffering personality profile in adulthood, which, in turn, contributes to problematic alcohol use (β = .21, *p* < .05); 4) childhood sexual victimization contributes (β = .18, *p* < .05) to the development of an anxious-suffering personality profile in adulthood, which, in turn, contributes (β = .21, *p* < .05) to problematic alcohol use; and finally, 5) childhood physical victimization directly contributes (β = .31, *p* < .01) to problematic drug use in adulthood. In other words, childhood psychological or sexual victimization contributes to the development of an anxiety-suffering personality profile, which subsequently contributes to the development of paraphilic sexual fantasies and problematic alcohol use in men who commit sexual aggression against women. In contrast, childhood physical victimization contributes only to the development of problematic drug use.

## Discussion

The primary objective of the present study was to empirically test the model proposed by Maniglio (2011), which posits that different forms of childhood victimization (sexual, psychological, physical) contribute to the development of an anxiety-suffering personality profile (e.g., anxiety, depression, loneliness), which, in turn, contributes to the emergence of paraphilic sexual fantasies as a coping strategy in men who have committed sexual aggression. Furthermore, because men who have committed sexual aggression may use various types of coping strategies, we also decided to test whether adding other types of coping strategies (alcohol and drug use) to Maniglio’s (2011) model would improve the model’s predictive power. Thus, this study examined whether childhood victimization is a risk factor for an anxiety-suffering personality profile which contributes to the development of both paraphilic sexual fantasies and problematic substance use in men who have committed sexual aggression.

### The initial model of Maniglio (2011)

The first model that we have identified, which is based on the initial model proposed by Maniglio (2011), was composed of four manifest variables and of one latent factor (composed of three manifest variables), and resulted in the identification of two trajectories: 1) childhood sexual victimization is associated with the development of an anxious-suffering personality type which, in turn, contributes to the emergence of paraphilic sexual fantasies; 2) childhood psychological victimization is associated with the development of an anxious-suffering personality type, which, in turn, contributes to the emergence of paraphilic sexual fantasies. These trajectories largely confirm Maniglio’s (2011) model, and also are consistent with previously reported personality profiles of sexual aggressors who have paraphilic sexual fantasies (violent, sadistic) (e.g., Brittain, 1970; Curnoe & Langevin, 2002; Gauthier, 2022; MacCulloch et al., 1983; Proulx & Beauregard, 2014), and are characterized by social isolation and low self-esteem. They also provide additional support for the argument, advanced in previous studies (e.g., Cortoni & Marshall, 2001; Ward et al., 2006; Ward & Hudson, 2000), that paraphilic sexual fantasies play an important role in regulating negative affective states (e.g., anxiety, depression, loneliness) in men who have committed sexual aggression.

### The Model With Other Inadequate Coping Strategies

The second model that we have identified, which includes other types of inadequate coping strategies (alcohol and drug use), was composed of four manifest variables and of three latent factors (each composed of three manifest variables), and resulted in the identification of five trajectories: four indirect trajectories contributing to the development of paraphilic sexual fantasies and problematic alcohol use, and one direct trajectory contributing to the development of problematic drug use. These trajectories largely confirm Maniglio’s (2011) model, and also partially support previous results that indicate that childhood victimization is associated with the development of other maladaptive self-regulation strategies for negative affects.

Of the five trajectories identified, two are the same as those identified in the first model. While childhood sexual and psychological victimization favor the development of paraphilic sexual fantasies mediated by an anxiety-suffering personality profile, the same cannot be said for childhood physical victimization for both models. Indeed, unlike Maniglio’s (2011) model, we found that childhood physical victimization contributes to problematic drug use, but to neither an anxious-suffering personality nor paraphilic sexual fantasies. Because childhood physical victimization is reported to be intimately related to the development of an antisocial and narcissistic personality in adulthood (Bernstein et al., 1998; Bierer et al., 2003; Cohen et al., 2014; Hengartner et al., 2013; Kounou et al., 2015; Krastins et al., 2014) and individuals with an antisocial personality are more likely to use drugs (Brennan et al., 2017; Evren et al., 2006; Mueser et al., 2006; Taylor & Lang, 2006), we hypothesize that the increased substance abuse observed in this study’s participants is due to the development of an antisocial and narcissistic personality profile, which is associated with thrill-seeking, such as the use of stimulant substances (e.g., cocaine).

In addition to the two trajectories contributing to the emergence of paraphilic sexual fantasies, two trajectories contributing to the development of problematic alcohol use were identified: 1) childhood sexual victimization contributes to the development of an anxious-suffering personality type which, in turn, contributes to the development of problem drinking; and 2) childhood psychological victimization contributes to the development of an anxious-suffering personality type which, in turn, contributes to the development of problem drinking. Both of these trajectories are consistent with several explanatory models of sexual aggression that suggest that substance use by men who commit sexual aggression is a strategy for emotional self-regulation. For example, Ward and colleagues (Ward et al., 1998, 2004) argue that men who have committed sexual aggression following the active avoidance path of the SRM model (i.e., actively seek to avoid acting out) adopt inadequate and ineffective coping strategies such as alcohol use.

In sum, this model indicates that childhood victimization is associated with the psychological development (personality profile) of men who have committed sexual aggression against women, and the development of inadequate strategies (paraphilic sexual fantasies, problematic alcohol and drug use) to cope with emotional distress (anxiety, loneliness, depression, low self-esteem).

### Theoretical Implications

The results of the present study provide insight into the developmental history of men who commit sexual aggression and their reliance on paraphilic sexual fantasies and alcohol and drug use. The trajectories identified suggest that the strategies adopted to cope with negative emotional states are shaped by specific combinations of developmental (victimization) and psychological (personality profile) factors that vary from one strategy to another. Also, the multiplicity of trajectories identified highlights the fact that there is a heterogeneity of strategies that men who commit sexual aggression rely upon to manage their negative affective states.

### Clinical Implications

The present study indicates that men who with an anxiety-suffering personality profile and who commit sexual aggression rely upon paraphilic sexual fantasies and alcohol consumption—both of which are risk factors for coercive sexual behaviors—to manage their negative affective states. These results highlight the importance of treatment programs for men who have committed sexual aggression, including a module that focuses on the development of appropriate and effective emotional regulation strategies. Therefore, the focus should be not only on managing paraphilic sexual fantasies and alcohol use, but also on developing strategies to manage negative emotional states without reliance on paraphilic sexual fantasies and alcohol. Furthermore, as highlighted by Turner-Moore and colleagues (2023), men who have committed sexual assault also exhibit non-paraphilic sexual fantasies. Therefore, therapists should also work with these men to implement their fantasies with the aim of replacing their paraphilic sexual fantasies with these non-paraphilic ones. Finally, therapists should consider incorporating group CBT techniques into their treatment programs, as they have shown promise for men who have committed sexual offenses and exhibit paraphilic sexual fantasies (Allen et al., 2020).

### Limitations

There are some limitations to this study. The first limitation concerns the composition of the sample. As the sample was exclusively composed of men who have committed sexual aggression against women, the scope of our results is limited to only that type of sexual aggression. As a result, the trajectories identified cannot be generalized to all men who have committed sexual aggression and have paraphilic sexual fantasies or problematic alcohol or drug use. It is also important to note that the identified trajectories cannot be generalized to men who have not engaged in coercive sexual behaviors but have paraphilic sexual fantasies or problematic alcohol or drug consumption. The second limitation of our study concerns the way paraphilic sexual fantasies were measured, namely as dichotomous variables that did not take into account the fantasies’ frequency, intensity, and nature. The consequence of this second limitation is that it is impossible to say whether the trajectories identified are relevant to all types of paraphilic sexual fantasies or only those associated with sexual coercion. However, it is possible that participants in this study, incarcerated men convicted of committing one or more sexual crimes, reported experiencing childhood abuse to try to justify their criminal behavior and the presence of paraphilic sexual fantasies. Furthermore, these men, who exhibit personality disorders such as borderline personality disorder, may retrospectively interpret childhood events as abuse, even though they were not actually abusive. Finally, it is important to note that one in five participants had committed a sexual homicide. This could have potentially impacted our results, especially regarding the presence of paraphilic sexual fantasies. We qualify this limitation as potential, given that several studies have reported that men who have committed a sexual homicide share many similarities and very few differences with men who have committed non-lethal sexual aggression (Nicole & Proulx, 2007; Oliver et al., 2007).

### Future Research

Given that men who have committed sexual aggression against women are known to differ from men who have committed sexual aggression against children in several aspects, notably developmentally (e.g., types of childhood victimization experienced) and psychologically (e.g., personality profile) (James & Proulx, 2020), future research should examine whether the trajectories that this study identified in men who have committed a sexual aggression against women are also present in men who have committed sexual aggression against children or men. Also, future studies should examine the emergence, development, and role of other coping strategies (e.g., problematic pornography consumption, compulsive masturbation, pathological gambling) relied upon by men who have committed sexual aggression.

## Note

1. In the context of this article, for the purpose of avoiding stigmatization, we have chosen to use the term “paraphilic sexual fantasies” instead of “deviant sexual fantasies.” However, it is important to note that the content of these paraphilic sexual fantasies refers to coercive sexual behaviors towards women or children, depending on the type of victim considered.
